# Long-term results of cochlear implantation in children with congenital single-sided deafness

**DOI:** 10.1007/s00405-020-06409-6

**Published:** 2020-10-20

**Authors:** Ann-Kathrin Rauch, Susan Arndt, Antje Aschendorff, Rainer Beck, Iva Speck, Manuel Christoph Ketterer, Till Fabian Jakob, Frederike Hassepass

**Affiliations:** Department of Otorhinolaryngology, ENT Clinic, Medical Centre, University of Freiburg, Freiburg, Germany

**Keywords:** Cochlear implant, Single sided deafness, Hearing rehabilitation, Age of cochlear implantation, Binaural hearing, Congenital cytomegalovirus infection, Cochlear nerve deficiency

## Abstract

**Purpose:**

The purpose of this retrospective study was to investigate the outcome and critical age of cochlear implantation in congenital single-sided deafness (SSD).

**Methods:**

11 children with congenital SSD were implanted with a cochlear implant (CI). Auditory performance was measured through the results of speech discrimination, subjective assessment by the Categories of auditory performance (CAP) score, the Speech, Spatial and Qualities scale questionnaire (SSQ) and the German version of the IOI-HA [Internationales Inventar zur Evaluation von Hörgeräten (IIEH, version for CI)].

**Results:**

Long-term follow-up [median: 3 years and 5 months (3;5 years)] revealed that nine children use their CI (> 8 h/day) and two became nonusers. In children aged below 3;2 years at surgery, there was a substantial long-term increase in speech discrimination and subjective benefit. Children over 4;4 years of age at CI surgery improved partially in audiological/subjective measurements. Among children above 5 years, the SSQ score did not improve despite further slight improvement in speech discrimination long-term.

**Conclusion:**

Our data suggest a critical age for CI surgery below 3 years in children with congenital SSD for successful hearing rehabilitation. It is mandatory to identify children with SSD as early as bilaterally deaf children.

## Introduction

Indication for cochlear implantation in single-sided deafness (SSD) has been established for adults with a reduction in tinnitus burden, benefit in localisation, speech discrimination in noise and quality of life [1–3]. Congenital SSD children were shown to perform lower compared to normal hearing (NH) peers at school and they more often had concomitant language impairment [4]. In congenital SSD children, however, no clear indication criteria for a critical age at surgery exist. Reimbursement for costs of implantation are accepted from health insurances in some countries, but there is no exact “cut-off” age for implantation. Some existing case studies and case series describe the short-term outcomes; but there are very little long-term data [2, 3, 5, 6]. To better advise patients, parents and caregivers, it is necessary, first, to consider both risks of surgery as well as rehabilitation/motivation efforts and, second, to relate them to realistic expectations and outcomes after cochlear implantation [7].

The time frame for maturation of the auditory pathway with binaural processing is assumed to lie just below 4 years [8–12]. Evidence exists for an “aural preference syndrome” in which the developing auditory pathway in SSD children reorganises with dominance towards the NH ear and weaker central representation of the hearing-impaired ear, involving areas for spatial processing [9, 13–16]. In detail, the sensory deprivation of the hearing-impaired ear leads to deterioration of localisation and sound integration abilities [17]. Representation of the deaf ear does not entirely vanish, but with prolonged period of deafness integration of new auditory input to the deaf ear changes due to central reorganisation and crossmodal plasticity [9, 12]. These changes may be long-lasting and not reversible in development. Therefore, from a neurophysiological perspective, it is necessary treating SSD with CI as early as possible to provide the benefits of binaural hearing.

Datalog analysis by Polonenko et al. revealed that pediatric CI SSD patients use their CI on average 7.1 up to 7.4 h a day [15, 16], showing their general acceptance of the CI.

Regarding the age of implantation, a current literature review of the literature demonstrates that there is a widely accepted recommendation for cochlear implant surgery before 42 months in order to prevent aural preference and crossmodal plasticity [13, 14]; the best outcomes are expected if surgery takes place within the first 36 months of the child’s life [12]. However, Thomas et al. found significant audiological and subjective benefit also in children implanted after 3 years and 6 months (3;6 years, up to 11;0 years). From a group of 21 congenital SSD children, eight were implanted above 4 years and had significant improvement in speech comprehension in noise and lateralisation ability with cochlear implant (CI) [8]. In assessing CI candidacy in congenital SSD children, it must be noted that unaided congenital SSD children learn to adapt to auditory cues and at least partly compensate the deficits of the deaf ear. These compensation efforts can be lost if congenital SSD children have used the CI for the first years and then became non-users [7].

The primary objectives of the present study were as follows: follow-up with pediatric congenital SSD patients after cochlear implantation to observe long-term outcomes: (1) audiological improvement, (2) subjective benefit (parents/caregivers and children), (3) identify long-term non-users, and (4) identify a critical age for CI implantation in SSD children by comparing different age groups.

## Methods

### General aspects

The setting of our retrospective study was a tertiary academic referral centre in a single-institution setup. This study was approved by the Ethics Committee of the University of Freiburg (proposal no. 03/17, operation number 191096, DRKS00020801) and conducted in accordance with the guidelines of the Declaration of Helsinki (Washington, World Medical Association, 2013). All parents/caregivers signed informed consent forms. The outcomes of some patients were discussed in previous studies [6, 18].

### Study subjects

Selection criteria for participation of the pediatric patients in this retrospective analysis were normal hearing (pure-tone average (PTA) < 20 dB in air conduction, auditory brainstem response thresholds (ABR) ABR = 20 dB in 10, ABR = 30 in 1, Table 1) on one ear and profound hearing loss on the other ear (PTA > 90 dB in air conduction; Table 1). When PTA was not appropiate in younger children, behavioural audiometry was done with a wobble tone between 0.25 and 8 kHz complemented by auditory brainstem response (ABR) and electrocochleography. Testing varied due to the age of the patients.

**Table 1:** Characteristics of subjects

Age group	Subject	Age at diagnosis (NHS or years; months)	Age at surgery (years; months)	Etiology	Follow-up period (years; months)	ABR of the SSD ear [dB]	ABR of the normal hearing ear [dB]
1	1	NHS	1;9	Unknown	4;3	> 90	20
	2	NHS	1;10	CMV	3;3	> 90	20
	3	NHS	3;0	Hypoxia	3;4	> 90	30
	4	2;6	3;2	CMV	4;10	> 90	20
2	5	NHS	4;4	CMV	4;5	> 90	20
	6	NHS	4;8	EVA	2;10	> 90	20
	7	NHS	5;0	Unknown	3;9	> 90	20
3	8	NHS	5;2	Unknown	3;5	> 90	20
	9	NHS	5;3	Unknown	1;9	> 90	20
	10	4;5	6;8	Ototoxic	3;2	> 90	20
	11	0;4	13;10	CMV	4;1	> 90	20

*NHS* newborn hearing screen, *CMV* cytomegalovirus, *EVA* enlarged vestibular aqueduct syndrome, *SSD* single-sided deafness

Prior to CI surgery, all children underwent a pre-examination: patient history, prior hearing assessments and outcome of the newborn hearing screening (NHS) were documented. Sensorineural hearing loss (SNHL) was assessed through subjective audiometry with age-appropriate testing comprising hearing thresholds and speech recognition of monosyllables. All subjects received a high-resolution computed tomography (HR-CT) of the temporal bone and a cranial magnet resonance imaging (MRI). None of the included subjects revealed pathological anatomical findings, except for enlarged vestibular aqueduct (EVA) syndrome in one child (subject 6). If further developmental disorders were known/suspected, the children underwent a neuropediatric examination before implantation, revealing no abnormalities. All patients/parents had > 2 months of decision time before signing informed consent for the study. They had been informed on the ramifications for daily life (e.g. localisation abilities), availability of bone conduction hearing systems and contralateral routing of signals (CROS) and assistive technologies such as frequency modulation (fm) devices before CI surgery, and about the necessity for participation in a mandatory rehabilitation process.

### Surgery and hearing rehabilitation

All subjects were implanted by two experienced surgeons. 4–6 weeks after surgery and successful wound healing, the initial activation of the CI was carried out. Interdisciplinary rehabilitation for cochlear implant surgery was implemented in an in-patient-setting every 3–4 months with technical fitting, logopedic therapy, music therapy and psychological counseling. Audiological and assessments by speech/language therapists were carried out on all rehabilitation dates. The direct audio link to the speech processor (SP) was used to practise the implanted ear exclusively. Auditory practise was gradually increased, according to developmental stage of the child and his or her CI experience, from detection of sounds to vocals, consonants and listening in noise, music and localisation tasks, at the beginning of rehabilitation supported by visuals. Very young childs above 2 years of age were trained to react in an age-appropiate game at auditory stimuli.

### Assessment of outcomes

#### Average hearing threshold of normal hearing (NH) ear and speech audiometry

PTA at 0.5, 1, 2 and 4 kHz (PTA4) or behavioural audiometry in free-field with wobble tone and open speech audiometry in free field were performed in a sound-proof chamber using a standard clinical set-up. Clinical standard tests were selected according to the age and cooperation of the child ranging from the Mainzer (contains multisyllables) and Göttinger speech test (a simplified monosyllabic test) to the Freiburger (monosyllabic test). Noise masking of the NH ear was not accepted by most subjects with young age and replaced by ear plugs.

#### Categories of auditory performance (CAP)

The CAP score (Archbold et al. [19]) was used to describe the discrimination ability of the implanted ear and was determined retrospectively by the assessment of the speech/language.

#### Speech, Spatial and Qualities of Hearing Scale (SSQ)

Subjective benefit was evaluated with the adapted SSQ by Galvin [20] and Galvin and Noble [21] which comprises two versions: a self-assessment for children and one for parents/caregivers [20]. The questionnaire asks for benefit in (1) speech, (2) spatial hearing and (3) quality of hearing in general listening conditions on a rating scale of 1–10 points (1:worst, 10:best, or “not applicable”).

#### International Outcome Inventory for Hearing Aids (IOI-HA) and CI use

We employed the German version of the IOI-HA [22], which has been adapted for CI users, to investigate different domains of benefit. The IOI-HA has a scale from 1 to 5 (1: least benefit; 5: most benefit) and seven items on CI use, benefit, and its impact on satisfaction, impairment and quality of life. As cutoff value for benefit from the CI for all questions 3 points per question were defined [23]. In addition, CI use was inquired by speech therapists in a standardised manner through patients’ and parents’/caretakers’ reports and declaratively assessed.

### Definition of age groups

As outcomes varied with age, three age categories were defined for better clarity (Table 1): age group 1 (four subjects: 1–4, up to 3;2 years at surgery, age group 2 (three subjects: 5–7, aged between 4;4 and 5 years at surgery) and age group 3 (four subjects: 8–11, above 5;2 years up 13;10 years at surgery).

## Results

Until 2019, 72 children with SSD were screened in our tertiary academic referral centre/cochlear implant programme. In the cluster of SSD children, 36 had congenital SSD. From the 36 congenital SSD children, 18 had indication for a CI and and received the surgery. Of the 18 children without indication, 12 had cochlear nerve deficiency (CND) and six had an unclear cause of SNHL and/or were not implanted due to the long period of deafness or because they refused surgery.

In 2016, 11 children were included in this retrospective analysis. Ten of these patients were included in a previous study with short-term data [6]. The follow-up period in the present study was 1;9 up to 4;10 years. The median age of surgery was 56 months (SD ± 39.69 months), or 4;8 years (average age at surgery: 59.64 months). The median follow-up was 41 months (SD ± 10.23 months), i.e. 3;5 years (average 42.64 months). Most pediatric CI candidates (*n* = 8/11) with congenital SSD were identified through the bilateral newborn hearing screening (NHS); obligatory in Germany since 2009. The other three children were diagnosed at age 2;6 years, 4;5 years and 4 months (Table 1). They were classified as congenitally deaf, based on anecdotical reports of the parents and pedaudiometric testings. The deafness period was calculated from their birth to the age where they underwent surgery, since all subjects were diagnosed with congenital SSD.

Based on anecdotical reports of children and parents/care givers during check-ups in the rehabilitation centre, 9 of 11 subjects wore their speech processor (SP) on a daily basis; they requested the SP or noted it to be out of function (batteries empty, coil-off). Two SSD CI recipients became non-users over the course of the study (subject 5 and 11, after 3;6 and 1 years, respectively; Tables 1 and 2).

**Table 2:** Speech discrimination in quiet

Subject	Age at surgery (years; months)	Follow-up period (years; months)	CI usage time	Speech discrimination ipsilateral with CI at 65 dB SPL (hearing test)		
				First fitting	2016	2019
1	1;9	4;3	All day	30 (Mainzer I)	30 (Mainzer I)	80 (Mainzer I)
2	1;10	3;3	All day	Too young	50 dB (PTA4)	32.5 dB (PTA4)
3	3;0	3;4	All day	10 (Mainzer I)	20 (Mainzer I)	90 (Mainzer I)
4	3;2	4;10	All day	80 (Mainzer I)	0 (Göttinger II)	60 (Göttinger II)
5	4;4	4;5	Non-user	20 (Göttinger II)	20 (Göttinger II)	Non-user
6	4;8	2;10	All day	0 (Göttinger II)	30 (Göttinger II)	80 (Freiburger)
7	5;0	3;9	All day	0 (Göttinger II)	10 (Göttinger II)	45 (Freiburger)
8	5;2	3;5	All day	0 (Göttinger II)	0 (Göttinger II)	40 (Freiburger)
9	5;3	1;9	All day	0 (Göttinger II)	0 (Freiburger)	25 (Freiburger)
10	6;8	3;2	Max. 8 h	20 (Freiburger)	15 (Freiburger)	10 (Freiburger)
11	13;10	4;1	Non-user	0 (Freiburger)	0 (Freiburger)	Non-user

*CI* cochlear implant, *dB* decibel, *SPL* sound pressure level

### *Average hearing threshold (0.5, 1, 2, 4 kHz) of NH ear pre- and postoperative (Fig. **1**)*

**Fig. 1:**
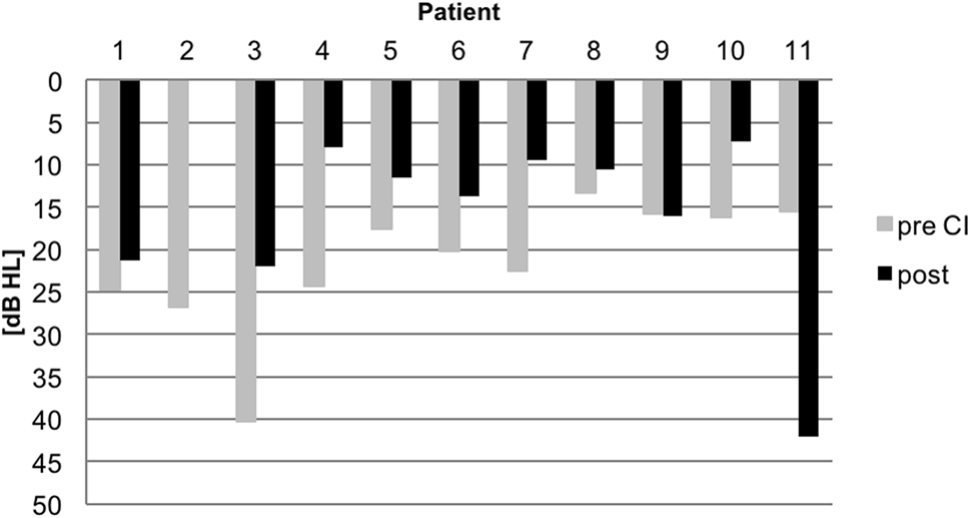
Average hearing threshold of NH ear pre- and postoperative. x-axis: subject number with pre- (grey bar) vs. postoperative (black bar) average hearing threshold, y-axis: hearing treshold (dB SPL)

There was no deterioration in average hearing threshold in the NH ear from pre- to postoperative measurements in subject 1–10. In subject 11 (non-user), PTA4 of NH ear deteriorated by 2019.

### *Speech discrimination in long-term follow-up (Table **2**)*

Because of the increasing age and developmental skills, different speech tests were employed during the follow-up. Therefore, intra- and interindividual comparison was difficult and statistical evaluation not applicable. Age group 1 improved clearly in speech discrimination tests or aided threshold measurements (subject 2). Age group 2 showed less benefit: subjects 5 aged 4;4 years became a non-user with best results of 2016 in Göttinger II. Due to age-appropriate testing subject 6 and 7 performed different speech tests during the follow-up (2016: Göttinger II vs. 2019: Freiburger). The latest results from 2019 demonstrated 80% (subject 6) and 45% (subject 7) in Freiburger indicating speech discrimination benefits. Age group 3 indicated none or only a slight improvement. Subject 11 became a non-user in 2018 (Table 2).

### *CAP results in the follow-up of 4 years’ time (Table **3**)*

**Table 3:** CAP scores

Subject	Age at surgery (years; months)	CAP 2016 (1–7)	CAP 2019
1	1;9	5	6
2	1;10	–	–
3	3;0	–	5
4	3;2	5	5
5	4;4	3	–
6	4;8	5	5–6
7	5;0	5	5
8	5;2	4	5
9	5;3	3	4
10	6;8	4	4
11	13;10	3	3–4

*CAP* categories of auditory performance

CAP scores and their development from 2016 to 2019 are shown in Table 3. Subject 3 was too young to assess the CAP score in 2016, but showed good development of speech in 2019 with a CAP score of 5, i.e. “understanding common phrases without lipreading”. Subject 5 had a CAP of 3 in 2016, “recognises environmental sources” without understanding speech and became non-user in 2019. Subject 11, already 13;10 years at surgery, started with a CAP of 3, which did not improve considerably in the course of the study, the subject became a non-user as well. All other subjects showed stable or improved CAP score by one point towards 2019. Across age groups, age group 3 had the lowest CAP scores in 2019 (median: 4; SD ± 0.63) vs. age group 2 (median 5.25; SD ± 0.35) and age group 1 (median 5; SD ± 0.58).

### Subjective evaluation with SSQ in long-term follow-up

**Fig. 2:**
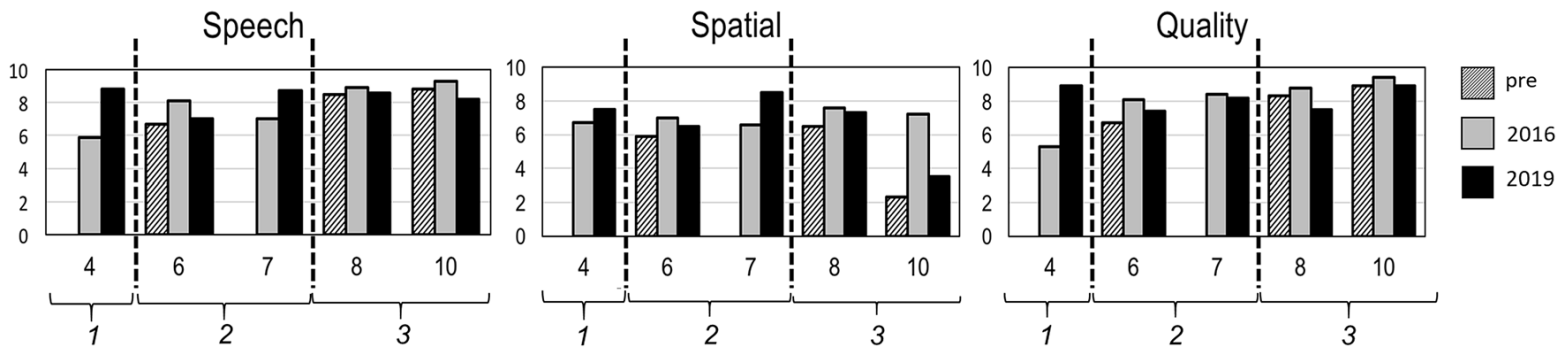
Speech, spatial and qualities scale questionnaire (SSQ) in children. On x-axis, the number of the subject from the study is plotted. On y-axis, the score from the SSQ questionnaire is stated. Legend on right side: time of data collection. The vertical dashed line shows distribution into the three different age groups (number in italics at horizontal axis): age group 1 (*n* = 1; subject 4), 2 (*n* = 2; subject 6–7) and 3 (*n* = 2; subject 8, 10). The youngest child (subject 4) showed the best outcome in all three sub-categories of the SSQ. Long-time data showed no progression in subjective benefit for the elder children with a duration of deafness above 5 years (subject 8, 10)

**Fig. 3:**
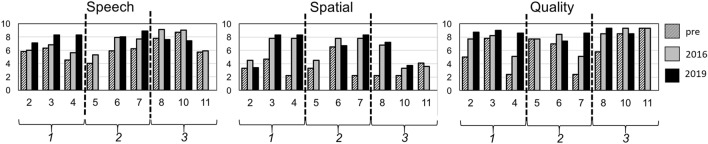
Speech, spatial and qualities scale questionnaire (SSQ) in parents. On x-axis, number of subject from the study is plotted. On y-axis, score from SSQ questionnaire is stated. See legend on right side for the time of data collection. The vertical dashed line shows distribution into the three different age groups 1–3 (number in italics at horizontal axis). SSQ by the parents showed a marked increase in subjective benefit in all three sub-categories for the youngest age category 1 of children up to 3 years and 2 months of age (*n* = 3; subjects 2–4). Age group 2 with subjects 5–7 (*n* = 3; age 4 years and 4 months up to 5 years) showed only a partial increase in subjective benefit and in age group 3 above 5 years of age (*n* = 3; subjects 8 and 10–11) there was no benefit

SSQ in childen could be obtained in 5 of 11 due to age (Fig. 2) and SSQ in parents in 9 of 11 CI recipients (Fig. 3). Through SSQ results, we could identify three groups of children with different outcomes (Figs. 2, 3; depicted by dashed line) and categorised them into the aforementioned age groups (Table 1).

### *SSQ in children (Fig. **2**)*

For all children, SSQ scores before implantation showed a median in the speech section of 8.5 (SD) ± 1.1; average score 8). In 2016, the median was 8.1 (SD ± 1.4; average score 7.8); in 2019, the median was 8.6 (SD ± 0.7; average 8.3), showing an increase in long-term median. In the spatial section, median scores also increased: the pre-surgery median was 5.9 (SD ± 2.3; average 4.9), in 2016 it was 7 (SD ± 0.4; average 7) and in 2019 it was 7.3 (SD ± 1.9; average 6.7). The hearing quality section did not show extensive changes. The pre-implantation median was 8.3 (SD ± 1.1; average 8), in 2016 it was 8.4 (SD ± 1.6; average 8) and in 2018 it was 8.2 (SD ± 0.7; average 8.2). Overall, subject 4 from age group 1 showed most progress in benefit across all subsections of the SSQ, while age group 2 (subject 6 and 7) showed less and age group 3 (subject 8, 10) the least benefit.

### *SSQ in parents (Fig. **3**)*

The best benefit in subjective results of all three subsections can be seen for age group 1. Age group 2 showed long-term improvement in the speech and spatial section, but not in the quality of hearing. Above 5 years of age (age group 3), there was neither short-term nor long-term improvement from the pre- to the postoperative stage. For age group 2, a partial long-term increase was mainly present in subsections speech and spatial. In the age group 3, a subjective benefit was nearly absent. SSQ scores before implantation across all subjects were median 5.9 (Standard deviation (SD) ± 1.5; average score 6.1), in 2016 6.8 (SD ± 1.4; average score 7) and in 2019 8.0 (SD 0.6; average 7.9). This showed a long-term increase. In the spatial section, median scores also increased long-term; pre-surgery median was 3.3 (SD ± 1.5; average 3.4), in 2016 6.8 (SD ± 2; average 6) and in 2019 7.2 (SD ± 2.1; average 6.6). Similarly, in the hearing quality section, median scores increased up to 2019: pre-implantation score was median 7.0 (SD ± 2.5; average 6.2), in 2016 8.2 (SD ± 1.6; average 7.7) and in 2019 8.6 (SD ± 0.6; average 8.6). Overall, age group 1 showed again the most significant benefit, followed by a partial long-term benefit in age group 2, whereas age group 3 had the least benefit.

### *IOI-HA-CI in children (Table **4**)*

**Table 4:** IOI-HA-CI in children in 2019

Subject	1 Use	2 Ben	3 RAL	4 Sat	5 RPR	6 Ioth	7 QoL	Sum	Av/Sum
2	5.0	4.0	2.0	5.0	4.0	4.0	4.0	28.0	4
3	5.0			5.0				10.0	5
4	5.0	5.0	4.0	5.0	5.0	5.0	5.0	34.0	4.86
6	5.0	5.0	4.0	5.0	3.0	3.0	5.0	30.0	4.29
7	5.0	5.0	4.0	5.0	5.0	5.0	5.0	34.0	4.86
8	5.0	3.0	3.0	5.0	5.0	5.0	5.0	31.0	4.43
10	5.0	4.0	2.0	5.0	2.0	4.0	2.0	24.0	3.43
Median	5.0	4.5	3.5	5.0	4.5	4.5	5.0	32.0	4.57
SD	0,00	0.82	0.98	0.00	1.26	0.82	1.21	5.1	0.73
Average	5.0	4.3	3.2	5.0	4.0	4.3	4.3	30.2	4.31

Mean score on each IOI-HA item for participants in three categories

*Use* hours of use per day, *Ben* benefit, *RAL* residual activity limitations, *Sat* satisfaction, *RPR* residual participation restrictions, *Ioth* impact on others, *QoL* quality of life, *Av/Sum* average result for one question

With respect to IOI-HA scores, data were obtained for 7 of 11 CI-users in 2019 (Table 4). Apart from subject 3, who answered two out of the seven items, all questions were answered by the subjects in 2019. Median IOI-HA-CI score was 4.57 (SD ± 0.73), on average 4.31 (SD ± 0.73). A score > 3 per item defined as benefit from CI was present in nearly all subjects. For item 1, all subjects gave the best result, equivalent to using the CI for more than eight hours per day. Item 2 (benefit) and 4 (satisfaction) had a result of median 4.5 (SD ± 0.82) and 5.0 (SD 0), respectively. With respect to positive impact on residual activities or hearing conditions—where subjects desired an improvement through the CI (item 3)—median was 3.5 (SD ± 0.98). Items 5 (residual participation restriction; median 4.5, SD ± 1.26), 6 (impact on others; median 4.5; SD ± 0.82) and 7 (quality of life; median 5.0, SD ± 1.21) were similarly improved. Results of age group 1 (subjects 2–4) was best with a total median score of 4.86 (SD ± 1.26, average 4.62), followed by age group 2 (subjects 6–7) with 4.57 (SD ± 0.4, average 4.57) and age group 3 (subjects 8, 10) with the least benefit of 3.93 (SD ± 0.71, average 3.93).

## Discussion

Our monocentric retrospective study aimed at a long-term evaluation of outcomes in pediatric congenital SSD CI recipients. Aside from examining their improvement in speech recognition and subjective benefit, it is essential to identify factors for successful hearing rehabilitation. This means, first and foremost, to identify the critical cut-off for the age of implantation, that is, the age until which children benefit from the intervention. The majority of the SSD children (*n* = 9 out of 11) in the follow-up after our initial study [6] continued to use their CI long-term, except for two subjects who became non-users. These subjects (subject 5, 11) showed no improvement in speech discrimination (Table 3). Benefit as stated in parents’ SSQ was mainly present in the speech and spatial section for subject 5 and there was no benefit in all three subsections for subject 11. Subject 5 was aged 4;4 years at implantation and additionally congenital cytomegalovirus (cCMV) infection might be the cause for the reduced benefit in hearing rehabilitation in this case. Subject 11 was, similar to children 8–10, late-implanted with an implantation age of 13;10 years.

### SSD children showed the best outcome below 3 years and 2 months at CI implantation: our findings in the context of current literature

In terms of speech discrimination, the youngest age group 1 (up to 3;2 years at CI surgery) showed the most progress in audiological and subjective outcome during the follow-up and they also had the best scores in 2019. Age-group 2 (4;4–5 years at CI surgery) showed an improvement in speech discrimination, but did not attain results as advanced as age group 1. Age group 3 (above 5;2–13;10 years at CI surgery) showed less benefit in speech discrimination. Accordingly, CAP scores were lowest for age group 3. In terms of the SSQ score, children in age group 1 benefited most from the intervention. Results of IOI-HA (CI) supported the findings from the SSQ: on average subjects benefited from the CI, were satisfied with the outcome and noted an improved impact. Median score per question in IOI-HA-CI was highest for age group 1 and lowest for age group 3. Parents/caregivers helped the children filling in the IOI-HA, which is why a possible influence on the results by them must be considered. Reduced benefit was seen in our study in age group 2 with benefit in SSQ regarding speech hearing and spatial hearing, but not in the quality of hearing, which might also be due to a saturation effect. Above 5 years of age, no subjective benefit—not even delayed—was seen for the subjects, despite speech the fact that discrimination improved. This points to the importance of identifying SSD children as early as possible to evaluate indication for CI surgery.

Supporting our findings, Távora-Viera and Rajan recommended implantation before the age of 4 years as they saw reduced results in speech understanding and localisation above this age [11]. Van Wieringen et al. reviewed the literature for implantation in SSD children and recommended implantation before 36 months of age [12]. They found that SSD children in particular lacked complex spoken language skills and it appeared that this does not resolve with increasing age without CI implantation [12]. Zeitler et al. reported about over 40% median correct scores in speech recognition and improvement in bimodal testing in noise in nine pediatric SSD CI patients aged 1.5–15 years (median duration of deafness: 2.9 years, 12-month follow-up) [24]. Although a direct comparison is not possible because of the different speech test batteries, we can demonstrate similar improvements in speech recognition in our study, especially for age group 1 (up to 80 and 90%, Mainzer I) and 2 (up to 45 and 80%, Freiburger). The oldest age group 3 scored poorest with speech recognition scores between 10 and 40% (Freiburger) in our study. Zeitler et al. studied 4 of 9 congenital SSD CI recipients with a mean deafness period of 6.5 years. Of the 4 children, 2 improved in word/sentence recognition score. These results should be interpreted with caution because of the low number of subjects (duration of deafness of 5.9 and 8.9 years), follow-up of 3 and 5 months and because of the lack of questionnaires on subjective benefit [24].

In contrast to our study, Thomas et al. described improvement in lateralisation ability and in speech discrimination in noise regardless of age, employing the German Oldenburg Sentence Test for Children (OLKiSa) for children implanted between 3;6 and 11;0 years, with the majority being implanted after age 3;6 years [8]. For bimodal speech perception thresholds, three patients were investigated. They reported that 4 of 21 subjects (aged 3;6/4;8/8;8/9 years) became limited or non-users [8]. Improvement in localisation was only seen if stimuli where presented from the CI side (lateralisation), which represents the improvement to overcome the head shaddow and not from front [8]. OLKiSa results (improvement: ≥ 1.5 dB) were available for 14 of 21 subjects, of which 3 of 14 improved in the condition sound/noise from front, 5 of 14 improved in the condition sound on NH ear/noise on SSD ear, and 7 of 14 improved in the condition speech on deaf ear/noise on NH ear [8]. Future studies should include speech comprehension testing in noise to better evaluate a possible “cut-off” age for benefit in SSD children. Due to the young age of the children and the difference in age groups and developmental stages, it is very difficult to obtain comparable results from identical speech tests, as evidenced by our and other studies [24, 25]. Nonetheless, age-appropiate testing is needed, resulting in different test batteries.

Our clinical findings are supported by histopathological observations of auditory pathway maturation in autopsied infants and neurophysiological studies [26, 27]. As maturation of the auditory system and especially binaural hearing is assumed to take place before the 4th year of life [9, 10, 14], CI implantation is strongly recommended before this threshold. Kral et al. recommended implantation before the age of 3.5 years [10]. Histopathological studies confirm this: maturation of the auditory pathway with myelinisation starts before birth and takes place approximately up to the 4th year of life [26]. This is supported by our findings as benefit from the CI decreased with rising age at implantation. Datalog analysis revealed a similar benefit in adult SSD patients compared to bilateral deaf patients in device usage, implicating subjective benefit [28]. IOI-HA questionnaire revealed usage times of more than eight hours among all children besides the 2 non-users in the present study. An evaluation of the datalog analysis could provide more accurate data in terms of usage times, especially for children with poorer speech recognition results. Datalog analysis by Polonenko et al. [15, 16] showed that pediatric SSD patients use their CI more than 7 h per day, implying a strong benefit of the binaural access to sound. Deep et al. showed that SSD children use their CIs on average 6.5 h per day (datalog analysis). In their study—and comparable to our results—the average age at implantation was 5 years (eight congenital and six acquired SSD) and speech recognition testing in 8 of 14 patients after 1 year of follow-up revealed a significant improvement in word recognition scores. Children younger than 4 showed most improvement and showed higher device usage time [25]. This is in line with our present findings revealing most benefit for children in age group 1 below 3;2 years.

Therefore, we recommend cochlear implantation in SSD children around 12 months of age comparable to bilaterally deaf children to best establish a basis for binaural hearing and prevent central reorganisation. If SSD children are diagnosed later, we recommend cochlear implantation before 3 years. Above this age, careful counseling of patients/parents/custodials is necessary. Further studies are needed, first, to identify the extent of crossmodal reorganisation and change in auditory responses in SSD children; second, and, especially with regard to congenital SSD, to identify the critical age for CI implantation; third, to identify the factors that cause these developments, as well as the extent to which they can be reversed upon CI implantation.

### Congenital SSD children: etiology, implications, auditory capacity of the NH ear and patient/parent counseling

In congenital SSD children, approximately at least 1/3–1/2 has CND [18], other sources state up to 40% in congenital/early-onset SSD and 28% in children with congenital asymmetric hearing loss [29].

In our study, 12 of 36 (1/3) of the screened children had CND, a lower percentage (33%) which is primarily due to a pre-selection of the children for the CI pre-examination. The high incidence of CND shows how important it is to carefully examine cranial MRI imaging before surgery. Furthermore, 4 of 11 of our congenital SSD CI recipients had a cCMV infection. The literature reports a percentage of > 20% cCMV infection as cause for congenital SSD [12, 30]. This emphasises the need to implement a nationwide cCMV screening. Hearing may deteriorate in cCMV infection [30]. Philips et al. reported that children with cCMV infection later tended to receive their CIs; they catched up with speech comprehension over a 5-year period, but lagged behind in terms of speech production compared to matched CI children with Connexin 26 (GJB2) gene-related deafness [31]. Beside the late implantation age, the cCMV infection could be the reason for the poor outcome of subject five in our study, implanted at 4;4 years who eventually became a non-user. SSD children should receive special monitoring of their NH ear as up to 75% (6/8) may develop delayed-onset contralateral SNHL [32]. Children with cCMV, in particular, bear the risk of developing learning difficulties in school due to difficulties with phonological working memory, pragmatic skills in social interactions and balance disorders [33]. The auditory reserve of the NH ear is relevant not only in children with cCMV etiology, but in all children with SSD: adult SSD CI patients showed significantly poorer hearing in the NH ear compared to an age-correlated group of NH subjects [34]. Our study revealed no deterioration in average hearing thresholds of the NH ear between the pre- to postoperative stage in 10 of 11 subjects (Fig. 1), with the exception of subject 11 with cCMV who became non-user. However, testing of NH ear varied due to age and improvement of test results through better cooperation of the children with increasing age, and possibly covering up smaller deterioration of hearing thresholds of the NH ear. This leads us to the counseling of patients/parents. SSD CI candidates must be advised on the possibility of progressive SNHL, reduced outcome with the CI at increasing implantation age and the necessity for the process of hearing rehabilitation. SSD children, implanted or not, must be supported during their education. No matter how good their compensation might be, deficits in speech discrimination and localisation abilities and increased listening effort must be taken care of. Alternatives should be discussed, e.g. CROS/BAHS systems, fm devices or digital remote wireless technologies. With increasing age, aspects of compensation ability and stigmatisation aspects become relevant in SSD children [7], important when considering late implantation. Stigmatisation may result in non-using of the CI, and in relevant psychological impairment and social isolation. Also, parental rejection of the intervention has to be taken into account during counseling [30].

### Limitations of the study

Limitations of our study are the retrospective design, lack of speech discrimination testing in noise for binaural benefit, evaluation of improved signal-to-noise ratio, difficult masking of the contralateral ear, and heterogeneous data regarding the speech audiometry because of different age and developmental skills of the children. Future studies should include evaluation of the childrens’ social performance and integration, stigmatisation and an analysis of datalogs to objectively assess the usage behaviour of SSD children as well as their exposition to different sound environments in SSD children. Examining the reaction of the children and auditory performance by turning the CI on/off could yield insights into the use, acceptance and benefit of the CI. Deep et al. tested speech recognition in noise and showed that SSD pediatric patients (*n* = 14, at least 1 year of device use, mean age at CI surgery 5 years) performed as well or better with the CI switched on versus off mode [25]. It cannot be excluded that subjects with implantation age above 5 years show a certain benefit, e.g. in noise, comparable to the report by Thomas et al. [8].

### Outlook: implications from our study and future demands for CI in pediatric SSD

For the first time, we can report on a considerable number of subjects with up to 4 years of follow-up post-operative. The best results were seen for age group 1. Above the age of 3;2 years, not only speech discrimination scores decreased, but also subjective benefit strongly decreased. Increase in speech discrimination still takes place after 1 year of hearing rehabilitation and highlights the importance of a long-term rehabilitation. Although a continued improvement was seen in late-implanted children over 3;2 years of age, our results show how important early CI implantation in SSD children is, given that the best results were obtained below this age. In our view, recommendation for implantation age is always a compromise between an age at surgery as old as possible and, with respect to auditory processing, an age as young as possible to avoid growing changes and central reorganisation. Based on our data, the data of previous studies and the knowledge on central reorganisation with potentially long-lasting changes in SSD, we recommend cochlear implantation in SSD children around the age of 12 months, and latest before the age of 3 years. Beyond this age, even more careful counseling is necessary as audiological and subjective benefit through the intervention may be absent.

As the number of implanted SSD children increases, implantation at an earlier age becomes more common. It should be our aim to identify congenital SSD children as early as bilaterally deaf children to achieve the best possible outcome with CI. Further prospective studies are needed to investigate long-term outcomes in speech discrimination in quiet/noise, localisation abilities, quality of hearing/life, tinnitus burden, stigmatisation, speech development and impact on educational/professional career.

## Conclusion

Congenital SSD children benefit from cochlear implantation before the age of 3 years. Up to this point, they can achieve a successful hearing rehabilitation of the deaf ear with measurable subjective and objective benefit. CI surgery for older congenital SSD children must be carefully and individually evaluated; however, they should not be categorically excluded from consideration. If they receive a CI, more intensive training post-surgery has to be recommended. Earlier identification of congenital SSD children is necessary, along with prospective study designs, to gain further insight into long-term outcomes and factors for successful hearing rehabilitation.

## Data Availability

Study data is available in the Department of Otorhinolaryngology, University Medical Centre Freiburg, Germany and will be stored in total for 10 years’ time.
